# miR-30 Family microRNAs Regulate Myogenic Differentiation and Provide Negative Feedback on the microRNA Pathway

**DOI:** 10.1371/journal.pone.0118229

**Published:** 2015-02-17

**Authors:** Martin G. Guess, Kristen K. B. Barthel, Brooke C. Harrison, Leslie A. Leinwand

**Affiliations:** Department of Molecular, Cellular and Developmental Biology and BioFrontiers Institute, University of Colorado, Boulder, Colorado, United States of America; Goethe University, GERMANY

## Abstract

microRNAs (miRNAs) are short non-coding RNAs that can mediate changes in gene expression and are required for the formation of skeletal muscle (myogenesis). With the goal of identifying novel miRNA biomarkers of muscle disease, we profiled miRNA expression using miRNA-seq in the gastrocnemius muscles of dystrophic *mdx4cv* mice. After identifying a down-regulation of the miR-30 family (miR-30a-5p, -30b, -30c, -30d and -30e) when compared to C57Bl/6 (WT) mice, we found that overexpression of miR-30 family miRNAs promotes differentiation, while inhibition restricts differentiation of myoblasts *in vitro*. Additionally, miR-30 family miRNAs are coordinately down-regulated during *in vivo* models of muscle injury (barium chloride injection) and muscle disuse atrophy (hindlimb suspension). Using bioinformatics tools and *in vitro* studies, we identified and validated *Smarcd2, Snai2* and *Tnrc6a* as miR-30 family targets. Interestingly, we show that by targeting *Tnrc6a*, miR-30 family miRNAs negatively regulate the miRNA pathway and modulate both the activity of muscle-specific miR-206 and the levels of protein synthesis. These findings indicate that the miR-30 family may be an interesting biomarker of perturbed muscle homeostasis and muscle disease.

## Introduction

Skeletal muscle can be affected by a variety of genetic and acquired pathologies, in most cases leading to a loss of muscle mass. In particular, Duchenne Muscular Dystrophy (DMD) is a lethal muscle degenerative disease that affects 1 in 3500 males [1] and is caused by mutations in the cytoskeletal protein dystrophin. While many therapeutic strategies are currently being developed to treat DMD (reviewed in [2]), the disease remains uncured. DMD is characterized by constant, contraction-induced myofiber degeneration followed by a regenerative response consisting of: activation of quiescent satellite cells, proliferation of the resulting myogenic precursor cells (myoblasts), and terminal differentiation and fusion of myoblasts into myofibers. Eventually, this regenerative response is exhausted leading to fibrosis, accumulation of adipose tissue, and progressive muscle loss and weakness (reviewed in [3]). Thus, a complete understanding of the formation of muscle (myogenesis) is critical to the development of diagnostic and therapeutic strategies.

microRNAs (miRNAs) are short, endogenous non-coding RNAs that post-transcriptionally repress gene expression by promoting translational repression and/or mRNA decay, resulting in decreased protein expression [4]. miRNAs have been shown to be essential for both embryonic myogenesis [5] and adult myogenesis after injury [6]. In addition to the finding that miRNAs as a whole are essential to proper muscle formation, individual miRNAs have been shown to play key roles in myogenesis, including species that regulate satellite cell quiescence (miR-489, [6]), promote proliferation (miR-133, miR-27 [7,8]), promote myoblast differentiation (miR-206, miR-1, miR-486 [9–11]), and regulate fiber type switching (miR-499, miR-208a, miR-208b [12]). While several studies have previously profiled miRNA expression in dystrophic human [13] and mouse muscle samples [14] using microarrays, advances in deep sequencing technology have increased the sensitivity, dynamic range, and reproducibility of miRNA expression profiling [15].

By deep sequencing small RNAs from wild-type C57Bl/6 (WT) and dystrophic *mdx4cv* gastrocnemius muscles, we found the miR-30 family miRNAs to be coordinately down-regulated when compared to WT. Given the high abundance in skeletal muscle and differential expression, we decided to further investigate the expression and function of miR-30 family miRNAs in mammalian skeletal muscle. Our results indicate that expression of the miR-30 family miRNAs is perturbed during alterations in muscle homeostasis *in vivo*, and that the miR-30 family miRNAs promote myoblast terminal differentiation and restrict proliferation *in vitro*. In addition, we identify the chromatin remodeling component *Smarcd2*, the transcriptional repressor *Snai2* and the miRNA pathway component *Tnrc6a* as direct miR-30 targets. Interestingly, we found that inhibition of *Tnrc6a* expression by miR-30 family miRNAs reduces the activity of muscle-enriched miR-206, indicating that the miR-30 family constitutes a negative feedback mechanism on the miRNA pathway. These findings expand our understanding of miRNA-mediated gene repression.

## Results

### miRNA sequencing reveals reduced miR-30 family expression in *mdx4cv* animals

**Fig 1:**
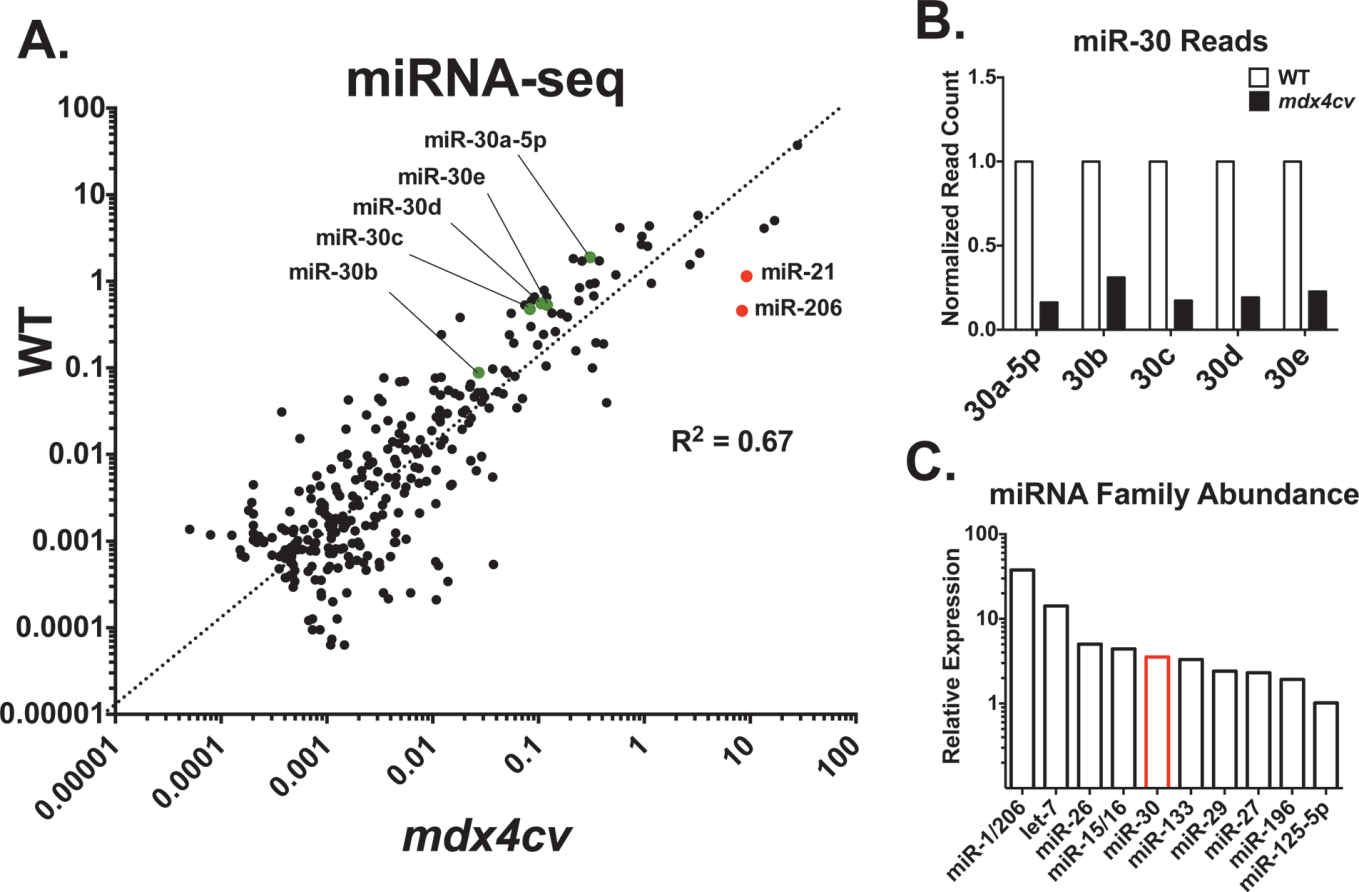
miRNA-seq reveals reduced miR-30 family miRNAs in mdx4cv muscles. RNA isolated from 3-month old C57Bl/6 (WT) and *mdx4cv* gastrocnemius muscles (n = 2/group) was adapter-ligated, reverse-transcribed, and PCR amplified to produce a cDNA library for sequencing using the Illumina platform. Following sequencing, reads were aligned and counted, then normalized to total miRNA reads. **(A)** Scatterplot shows the top 300 most abundantly cloned miRNAs. Note miR-206 and miR-21 (red, overexpressed in *mdx4cv*) and miR-30 family (green, down-regulated in *mdx4cv*). **(B)** Normalized read counts of miR-30 family miRNAs displayed relative to WT. **(C)** miRNA family expression from miRNA-seq experiment shows that miR-30 family miRNAs are the 5th most abundantly cloned, conserved miRNA family in skeletal muscle.

**Fig 2:**
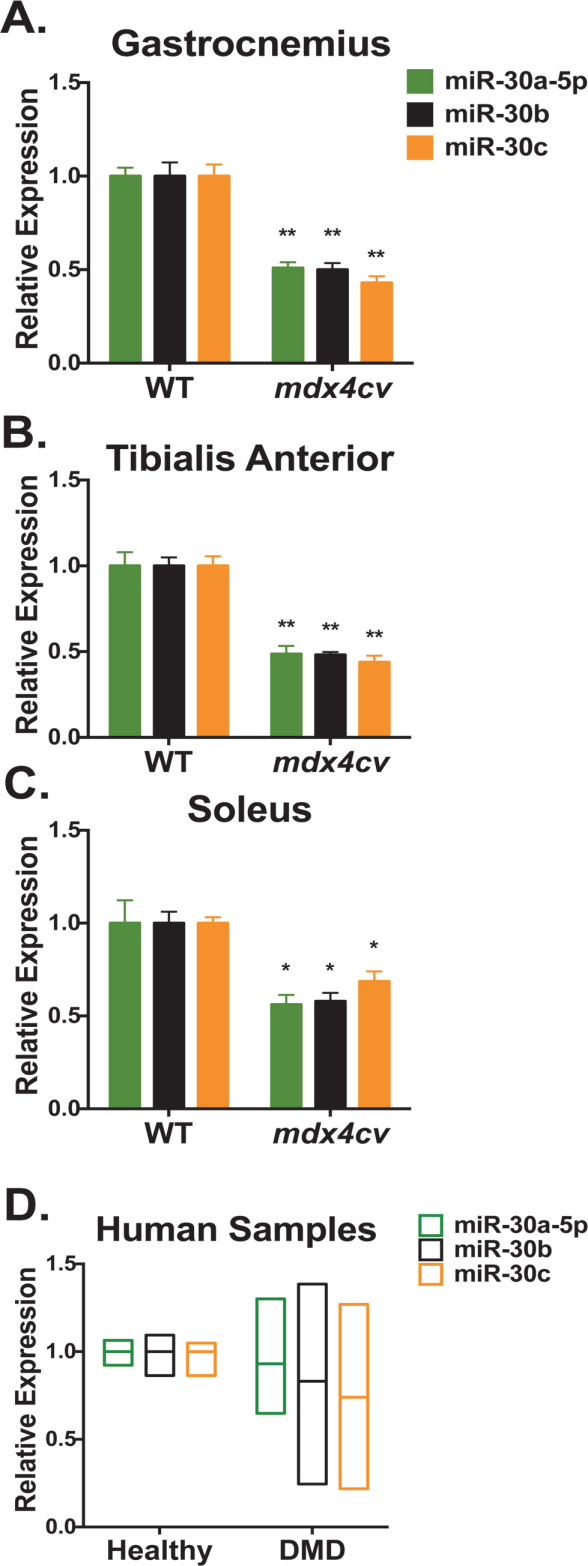
qRT-PCR validation of reduced miR-30 family miRNA expression. qRT-PCR analysis of RNA isolated from whole **(A)** gastrocnemius, **(B)** tibialis anterior (TA), and soleus **(C)** muscles of 3-month old WT and *mdx4cv* animals validates down-regulation of miR-30a-5p, miR-30b and miR-30c (miR-30a/b/c) by qRT-PCR. n = 4 animals/group, error bars = SEM, *P≤0.05, **P≤0.001 compared to WT. **(D)** qRT-PCR measurement of miR-30a/b/c in human patient biopsies relative to U6 snRNA.

In order to identify miRNAs that are dysregulated during muscle pathogenesis, we hypothesized that, as dystrophic muscle is undergoing constant cycles of degeneration/regeneration, miRNAs differentially expressed between dystrophic and healthy muscle may represent novel biomarkers of muscle homeostasis. We thus performed high-throughput small RNA sequencing (miRNA-seq) on RNA isolated from the gastrocnemius of 3-month old male WT and *mdx4cv* animals (Fig. 1A and S1 Table, n = 2 animals/group). Confirming our data set, we found that miR-206 and miR-21, which have been previously shown to be up-regulated in dystrophic mice [9,16], were up-regulated 18- and 8-fold, respectively. Interestingly, we also found that the normalized read counts for the entire miR-30 family were strikingly reduced in *mdx4cv* animals (Fig. 1B), and that the miR-30 family is the 5th most highly expressed miRNA family in skeletal muscle (Fig. 1C). The miR-30 family miRNAs belong to the same seed family and thus share identical seed sequences (S1 Fig.) and likely regulate an overlapping set of targets. While the miR-30 family includes 5 mature miRNAs (miR-30a-5p, miR-30b, miR-30c, miR-30d and miR-30e [NCBI: NR_029533, NR_029534, NR_029716, NR_029718, NR_029602]), for this study we have focused on miR-30a-5p, miR-30b and miR-30c (“miR-30a/b/c”) due to sequence similarity of miR-30a-5p, miR-30d and miR-30e (differing by only one nucleotide each) (S1 Fig.). In order to confirm these results, we performed qRT-PCR on a larger (n = 4) cohort of animals, and similarly found a marked reduction in the levels of miR-30a/b/c in the dystrophic gastrocnemius, soleus, and tibialis anterior (TA) muscles (Fig. 2). This reduction was least pronounced in the slow-twitch soleus muscle, where baseline miR-30 levels are lower than in the gastrocnemius and TA muscles (S2 Fig.). In human DMD patient biopsies, we did not observe a significant difference in miR-30a/b/c levels between healthy and diseased samples (Fig. 1D), but we did observe an increase in the variability of miR-30a/b/c expression, in agreement with the clinical heterogeneity of DMD patient biopsies [17].

### miR-30a/b/c expression decreases during acute injury and muscle disuse atrophy

**Fig 3:**
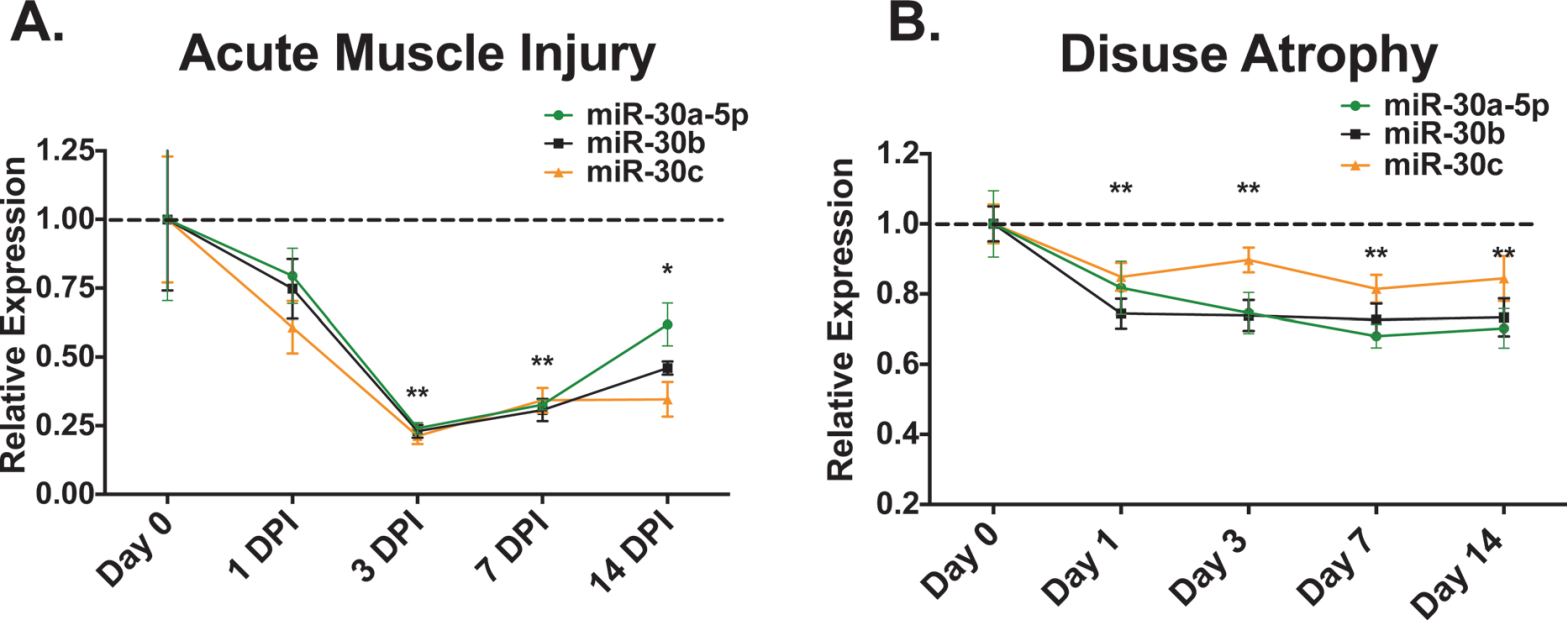
miR-30a/b/c are decreased after acute injury and muscle disuse atrophy. **(A)** qRT-PCR of miR-30a/b/c from BaCl_2_-injured WT gastrocnemius muscles. After reaching a minimum on day 3 post-injury (DPI), miR-30 levels begin to return towards uninjured levels on days 7 and 14. Expression displayed as injured (R) gastrocnemius relative to uninjured contralateral (L) controls. **(B)** qRT-PCR measurement of miR-30a/b/c during 14 days of hindlimb suspension. Expression displayed as miR-30a/b/c level in unloaded calf complex relative to contralateral control for indicated time point. Error bars = SEM, *P≤0.05, **P≤0.001 compared to contralateral controls for a given miRNA at the indicated time.

Given our observed decrease in miR-30 family expression in a pathological setting of constant degeneration/regeneration (*mdx4cv*), we wanted to examine miR-30 family expression in other models of skeletal muscle pathology, including regeneration after acute injury and muscle disuse atrophy. Accordingly, we first performed barium chloride injury in the gastrocnemius muscles of WT animals to test regeneration after injury *in vivo* and measured miR-30 family expression on 1, 3, 7 and 14 days post-injury (DPI) in comparison to uninjured contralateral controls. Barium chloride is an established model of muscle degeneration that induces widespread necrosis of myofibers and subsequent regeneration [18]. After injury, miR-30 family expression is reduced and reaches a minimum on day 3 post-injury (∼4–5 fold reduction in miR-30a/b/c) (Fig. 3A) corresponding to a time point at which the muscle is largely degenerating (S3 Fig.), indicating a correlation between low miR-30a/b/c levels and muscle degeneration.

Next, we measured miR-30 levels during hindlimb suspension-induced muscle disuse atrophy- an established model in which the unloaded limb undergoes a loss of muscle mass and strength, along with molecular adaptation including altered myosin heavy chain (MyHC) composition [19]. After beginning hindlimb suspension miR-30a/b/c levels decreased after only 1 day (Fig. 3B) and remained decreased throughout the 14 day experiment.

### miR-30a/b/c expression increases during myogenesis

**Fig 4:**
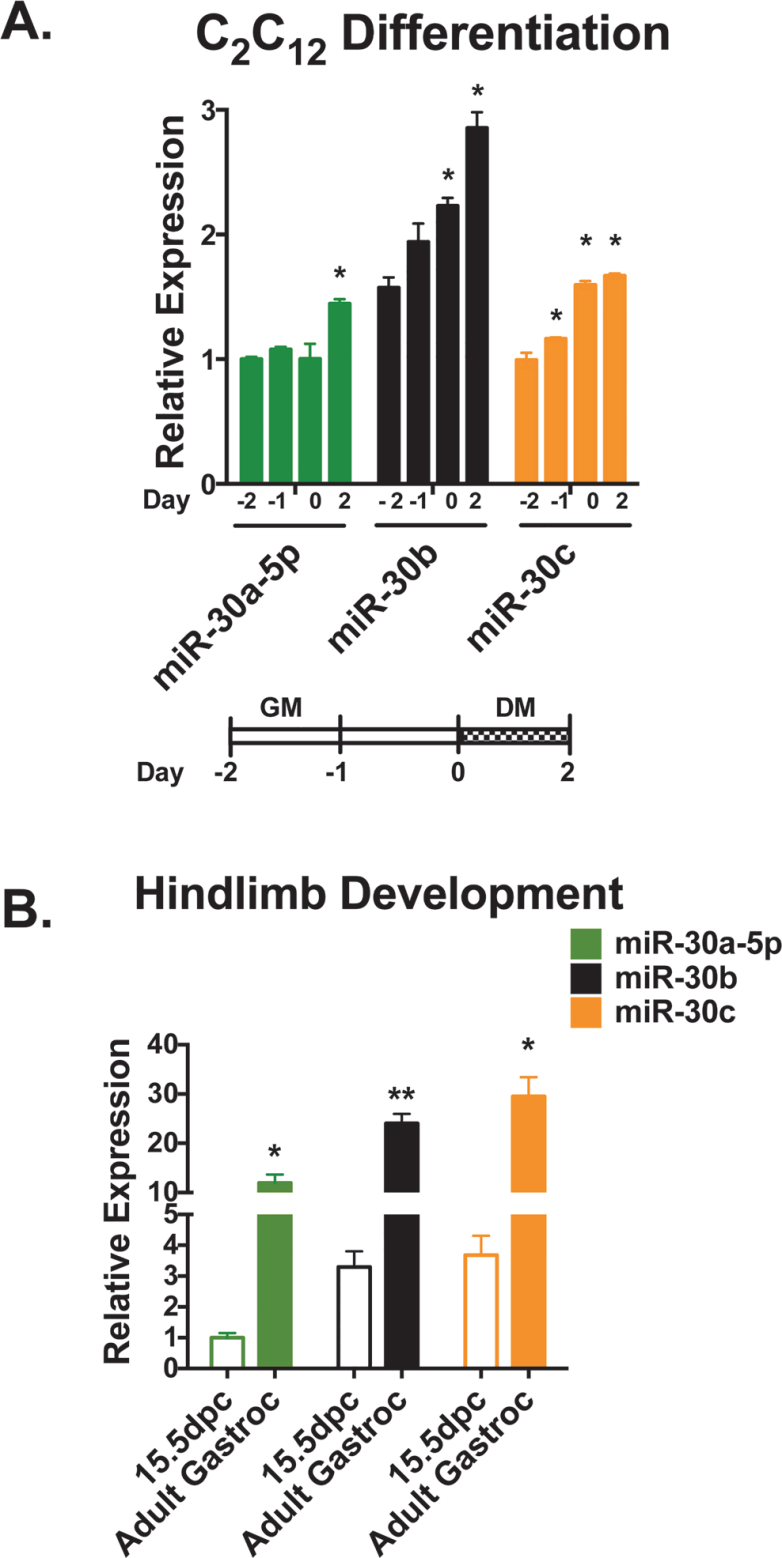
miR-30a/b/c expression increases during myogenesis. **(A)** Relative miR-30a/b/c expression levels measured using qRT-PCR indicate increased expression during C_2_C_12_ differentiation. Below, schematic indicating differentiation time course. GM = growth medium, DM = differentiation medium. Error bars = SEM, *P≤0.05 compared to day -2 for each miRNA. **(B)** qRT-PCR of miR-30 a/b/c from WT 15.5 days post-coitum (dpc) embryo limb buds indicates lower relative levels of expression than adult gastrocnemius muscles. miR-30 family expression displayed relative to 15.5 dpc miR-30a-5p levels. Error bars = SEM, *P≤0.05, **P≤0.001 compared to 15.5 dpc levels for given miRNA.

To test if the reduction in miR-30 family miRNA expression found in dystrophic, injured and atrophic muscle correlates with expression changes in myoblasts, we measured miR-30a/b/c expression during C_2_C_12_ myoblast differentiation *in vitro*. Following withdrawal of serum from the medium, we observed increases in miR-30a-5p (∼1.5 fold), miR-30b (∼2 fold) and miR-30c (∼2 fold) expression as differentiation progressed (Fig. 4A), indicating that the miR-30 family is expressed in myoblasts.

Given these dynamic expression changes during adult myogenesis *in vitro*, changes in miR-30 family expression could also be expected during developmental myogenesis. To test this, we isolated limb buds from WT 15.5 days post-coitum embryos and compared the levels of miR-30a/b/c expression to those in the adult gastrocnemius. Again we found high levels of miR-30a/b/c expression in the fully mature, adult tissue and low levels (∼10 fold lower on average for miR-30a/b/c) in the developing muscle (Fig. 4B), indicating that increased miR-30a/b/c expression is a feature of both adult and developmental myogenesis.

### miR-30 family miRNAs promote a myogenic program *in vitro*

**Fig 5:**
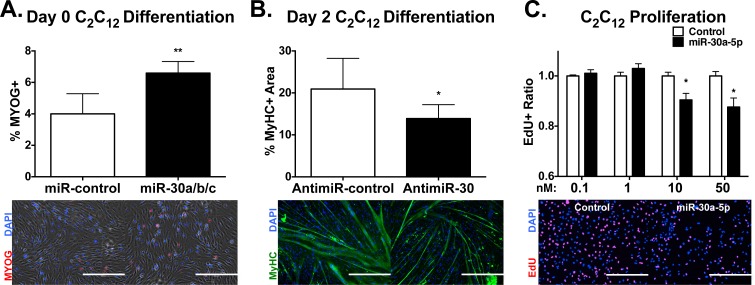
miR-30a/b/c promote differentiation of myoblasts *in vitro*. **(A)** Quantification of MYOG+ nuclei (n ≥ 9 images) in C_2_C_12_ myoblasts transfected with the indicated pre-miRs at 10nM final concentration. 24 hours after transfection, cells were stained with 4’-6-diamidino-2-phenylindole (DAPI) and anti-MYOG antibodies (red).**P≤0.001 compared to pre-miR-control cells, error bars = SEM. Below, representative images of fluorescence and brightfield microscopy. Scale bars = 200μm. **(B)** Quantification of % MyHC+ area (n ≥ 9 images) from C_2_C_12_ cells transfected with the indicated antimiRs at 10nM final concentrations. 48 hours following withdrawal of serum from the medium, cells were stained with anti-myosin heavy chain (MyHC) antibodies (green) and DAPI. *P≤0.05 compared to antimiR-control, error bars = SEM. Below, representative fluorescence images. Scale bars = 400μm. **(C)** Quantification of proliferating cells using 5-ethynyl-2’-deoxyuridine (EdU) analysis. 24 hours after electroporation of pre-miR-30a-5p and scrambled controls at indicated concentrations, C_2_C_12_ cells were pulsed with EdU for 2 hours, then stained for EdU incorporation and DAPI. *P≤0.05 vs. scrambled control at equivalent concentrations. Error bars = SEM Right, representative fluorescent images, scale bars = 200μm.

To gain insight into whether increased miR-30 family miRNA expression promotes or is merely correlated with myogenesis, we performed gain-of-function experiments in C_2_C_12_ myoblasts. Before transfecting cells and performing phenotypic analysis, we first optimized transfection and found that 24 hours following transfection of synthetic pre-miR-30a-5p, mature miR-30a-5p levels increased dose-dependently when compared to a scrambled pre-miR control at equivalent concentrations (S4 Fig.). Thus, we transfected C_2_C_12_ myoblasts with an equimolar mix of synthetic pre-miR-30a-5p, pre-miR-30b and pre-miR-30c at 10nM total concentration. Twenty-four hours after transfection, quantification of myogenin-positive (MYOG+) nuclei indicated a striking 65% increase (*P* = 5e-5)(Fig. 5A), indicating that the miR-30 family promotes terminal differentiation of myoblasts *in vitro*.

To test if the inverse is true, we utilized chemically modified, antisense oligonucleotides to inhibit miR-30 family function. Twenty-four hours after transfection with increasing dosages of antimiR-30, we found that antimiR-30 dose-dependently reduced miR-30a/b/c compared to an antimiR directed against a non-mammalian miRNA (antimiR-control) (S4 Fig.). As indicated by the percentage of MyHC+ area, 24 hours following transfection antimiR-30 restricted the differentiation of C_2_C_12_ myoblasts (Fig. 5B), again indicating that miR-30 family miRNAs promote myoblast differentiation.

During myoblast differentiation, multipotent progenitor cells commit to the myogenic lineage, proliferate, then withdraw from the cell cycle prior to fusion. We thus wondered whether ectopic miR-30 family miRNA expression could decrease the proportion of proliferating cells. To this end we transfected proliferating C_2_C_12_ with a representative synthetic miR-30 family member, miR-30a-5p, then performed 5-ethynyl-2’-deoxyuridine (EdU) proliferation analysis. In comparison to a scrambled pre-miR control at equivalent concentrations, EdU incorporation was reduced dose-dependently by 10% and 15% (*P* ≤ 0.05) in 10nM and 50nM transfected cells, respectively (Fig. 5C), indicating that high miR-30 family expression reduces the proportion of proliferating myoblasts *in vitro*.

### Putative direct miR-30 family targets include epigenetic, transcriptional, and post-transcriptional regulators of gene expression

**Fig 6:**
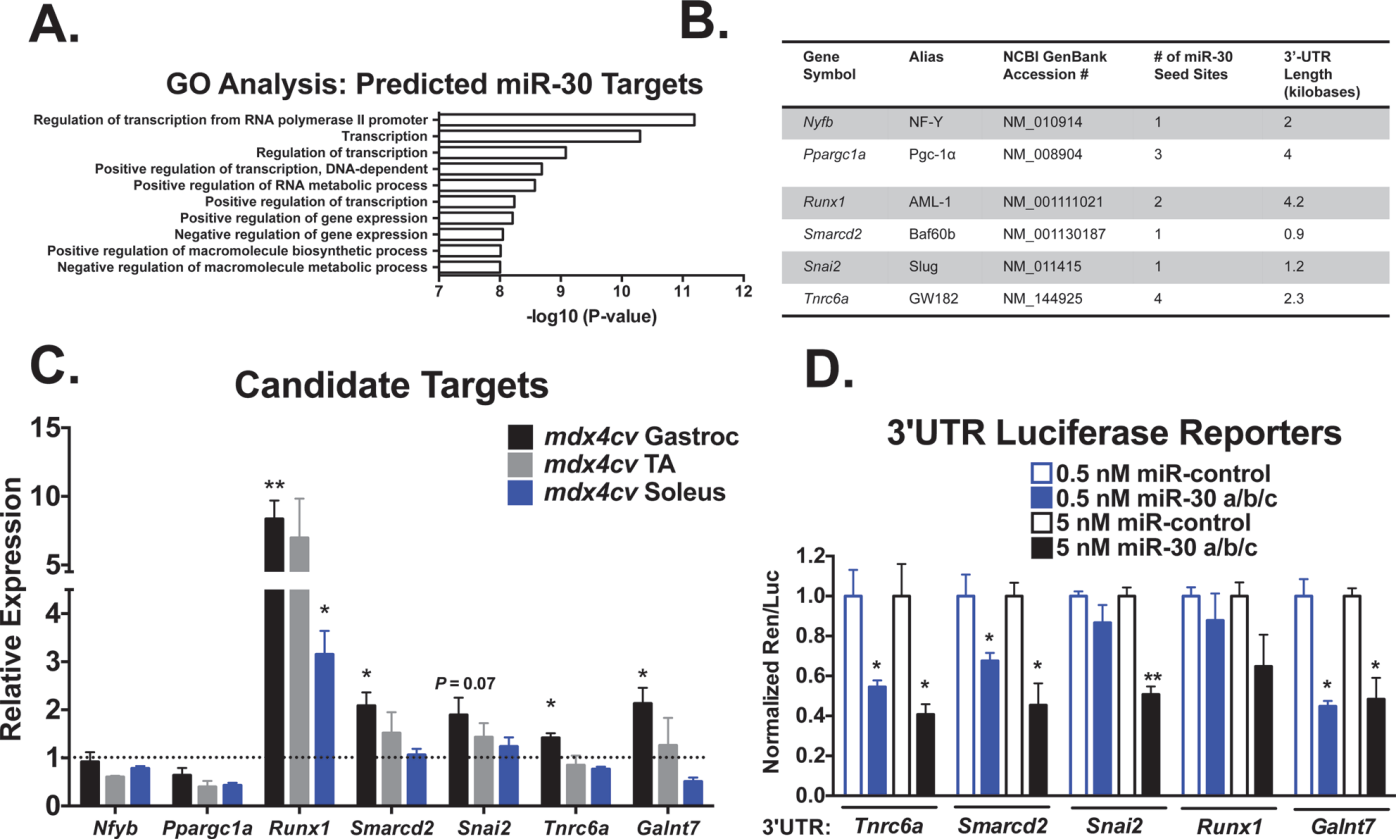
miR-30 family target identification and validation. **(A)** GO analysis of predicted miR-30 targets (TargetScan 6.2) is shown sorted by *P*-value for enriched biological processes. The most significant biological processes include regulation of transcription, gene expression, and macromolecule biosynthesis. **(B)** Candidate miR-30 targets. **(C)** qPCR measurement of candidate miR-30 targets in 3-month old *mdx4cv* tissue, relative to 18S. Error bars = SEM, *P≤0.05, **P≤0.001 compared to WT tissue. **(D)** 3’-UTR reporter assays of candidate miR-30 targets. Indicated concentrations of pre-miR-30a/b/c or pre-miR-control were transfected along with full-length 3’-UTR luciferase reporter constructs into C_2_C_12_ cells. *P≤0.05, **P≤0.001 compared to pre-miR-control transfected cells at equivalent concentrations. Error bars = SEM.

To identify direct miR-30 family targets, we first utilized TargetScan 6.2 [20] to identify predicted targets. To narrow the candidate target list as well as gain insight into the biological processes and pathways that may be regulated by the miR-30 family, we took the 1133 predicted targets and performed gene ontology (GO) analysis using the Database for Annotation, Visualization and Integrated Discovery (DAVID) [21]. When sorted for *P*-value, the functionally annotated biological processes that are most enriched in the list of predicted miR-30 family targets include the regulation of transcription, gene expression, and macromolecule synthesis (Fig. 6A). From these categories, we then selected 6 candidate genes that have been previously shown to be involved in myogenesis (Fig. 6B), including *Nfyb*, a subunit of the NF-Y transcription complex important in regulating proliferation during myogenesis [22], *Ppargc1a* (Pgc-1α), a transcription factor that regulates mitochondrial biogenesis in muscle [23], *Runx1* (AML-1) a subunit of the core binding factor (CBF) and transcriptional repressor of myogenesis [24], *Smarcd2* (Baf60b), a SWI/SNF chromatin remodeling complex subunit important in muscle differentiation [25], *Snai2* (Slug), a transcriptional repressor important in muscle differentiation [26], and *Tnrc6a* (GW182), an argonaute binding partner and key factor required for all miRNA-mediated repression [27].

To further sort these candidates, we measured the expression levels of their mRNAs in *mdx4cv* skeletal muscles by qRT-PCR, including *Galnt7* as a positive control miR-30 target [28]. If miR-30 directly regulates the expression of these candidates at the mRNA level, one could expect de-repression in *mdx4cv* muscles where miR-30 family expression is reduced. While no change was observed for *Nfyb* and *Ppargc1a*, we found that *Runx1, Smarcd2*, and *Tnrc6a* were increased in their expression in the gastrocnemius muscles and that *Snai2* trended towards an increase (*P* = 0.07) (Fig. 6C), indicating that these may be direct miR-30 targets. While *Nfyb* and *Ppargc1a* may still be regulated at the translational level, we relied on an initial screen of mRNA expression, reasoning that mRNA and protein repression is generally well correlated [29,30].

### *Tnrc6a, Smarcd2*, and *Snai2* are regulated by miR-30a/b/c

To validate direct regulation of *Runx1, Smarcd2, Snai2*, and *Tnrc6a* by miR-30a/b/c, we cloned the full length 3’-UTRs containing miR-30 target sites from C_2_C_12_ genomic DNA and inserted the fragments downstream of the *Renilla* luciferase coding sequence in psiCHECK-2. We then transfected these constructs into C_2_C_12_ cells along with synthetic pre-miR-30a/b/c or control pre-miR, and measured the luciferase signal following 24 hours in culture. Interestingly, we found the highest level of repression for *Tnrc6a*, with ∼50% and ∼60% reductions in activity for 0.5nM and 5nM pre-miR-30a/b/c, respectively (Fig. 6D). Additionally, we found significant (*P* ≤ 0.05) ∼50% reductions in luciferase activity for *Smarcd2* and *Snai2*, known negative regulators of the myogenic gene program, indicating that miR-30a/b/c may promote myogenesis through regulation of these factors.

### miR-30 regulates miRNA-mediated post-transcriptional regulation and protein synthesis

**Fig 7:**
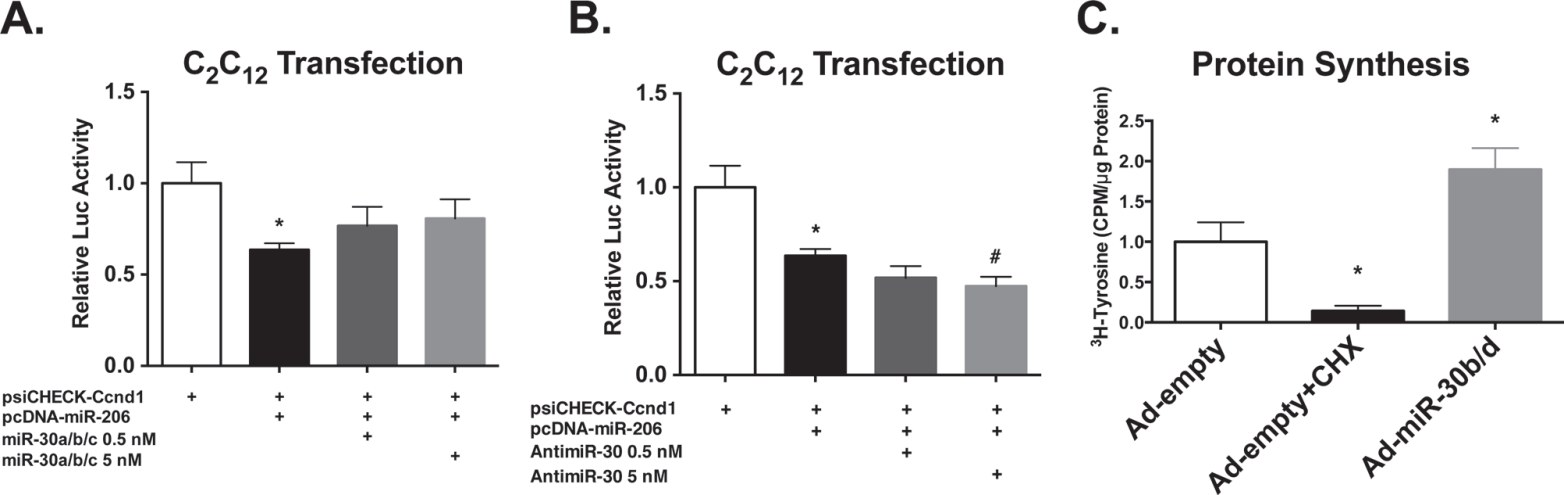
miR-30 regulates miRNA-mediated repression and protein synthesis. **(A)** Luciferase reporter activity of a *Ccnd1* 3’-UTR reporter during miR-206 and miR-30a/b/c overexpression. Addition of miR-30a/b/c relieves repression of the *Ccnd1* 3‘-UTR luciferase reporter by miR-206. *P≤0.05 compared to mock transfection. Error bars = SEM. **(B)** miR-30 inhibition enhances repression of the *Ccnd1* luciferase reporter by miR-206. *P≤0.05 compared to mock transfection, #*P*≤0.05 compared to pcDNA-miR-206 alone. Error bars = SEM. **(C)** Protein synthesis measured by ^3^H-tyrosine pulse labeling in C_2_C_12_ myotubes infected with a miR-30b/d-expressing adenovirus or empty virus control. Cycloheximide (CHX) included as a negative control. *P≤0.05 compared to empty adenovirus control. Error bars = SEM.

After finding that the *Tnrc6a* 3’-UTR was repressed ∼60% by miR-30a/b/c overexpression, we wondered if *Tnrc6a* repression may play a key role in regulating global miRNA-mediated repression. If miR-30 family miRNAs control miRNA repression by targeting *Tnrc6a*, we could expect that high levels of miR-30 would repress *Tnrc6a* levels resulting in global de-repression of miRNA targets and increased protein synthesis. To determine whether modulating miR-30 family miRNA levels affects miRNA repression, we tested the ability of muscle-specific miR-206 to repress a known target, cyclin D1 (*Ccnd1*)[31], during miR-30 family over-expression or inhibition. As expected, over-expression of miR-30a/b/c de-repressed *Ccnd1* luciferase reporter activity (Fig. 7A), and miR-30 family inhibition enhanced *Ccnd1* repression by miR-206 (Fig. 7B), showing that miR-30 family miRNAs can negatively regulate the activity of other miRNAs.

Another outcome of this hypothesis would be a general increase in protein synthesis in the presence of high miR-30 family miRNA levels, mediated by the de-repression of miRNA targets. In order to test this theory, we measured the levels of protein synthesis by performing a ^3^H-tyrosine incorporation assay on C2C12 cells infected with a miR-30b/d-expressing adenovirus. Indeed, after normalizing to protein content, we found a significant ∼2-fold increase (*P* ≤ 0.05) in ^3^H-tyrosine incorporation in miR-30b/d over-expressing myotubes when compared to controls (Fig. 7C), indicating that miR-30 family miRNAs promote high levels of protein synthesis, likely through de-repression of miRNA targets.

## Discussion

Here we show that the expression of miR-30 family miRNAs is dynamic in skeletal muscle pathologies, with low miR-30 being correlated with degeneration and muscle mass loss, and high miR-30 associated with myogenesis and protein synthesis. Numerous functions have been described for the miR-30 family, including regulation of fibrosis, apoptosis, and hypertrophy in cardiomyocytes [32–34], regulation of pronephros development in the kidneys [35], as well as the regulation of the epithelial-to-mesenchymal transition in hepatocytes [36]. Notably, little has been published about the expression and role of the miR-30 family in skeletal muscle.

In zebrafish, Ketley et al. recently showed that the miR-30 family promotes a fast muscle phenotype during embryonic muscle development and that inhibition of the miR-30 family in zebrafish embryos increased the percentage of slow fibers [37]. The authors attribute the observed disruption of embryonic myogenesis to regulation of the hedgehog transmembrane receptor *smoothened*, which when elevated results in abnormal patterning of muscle fibers [37]. While *smoothened* is not predicted to be a conserved miR-30 family target in mice, the possibility exits that miR-30 family miRNAs play a critical role in the regulation of embryonic muscle development and fiber type specification. Given that fast twitch fiber-types are preferentially affected in DMD [38], it is tempting to speculate that the decrease in miR-30 family expression in *mdx4cv* muscle is a compensatory mechanism to promote an increase in slow-twitch, fatigue resistant fiber types. Future studies will be needed to test this hypothesis *in vivo*. In another recent publication, Soleimani et al. proposed that miR-30-mediated regulation of the transcriptional repressor SNAI1 facilitates entry into the myogenic gene program and promotes differentiation of primary mouse myoblasts [26]. While these findings are in agreement with our observations of miR-30 family effects on proliferation and differentiation *in vitro*, we were unable to assess the expression pattern and function of miR-30 family members in non-muscle cell types *in vivo*. The possibility therefore exists that the expression changes we have observed are a composite of expression changes in myoblasts as well as endothelial, immune, and fibro-adipogenic cell types *in vivo*. Identifying the cell-type specific expression pattern of the miR-30 family in WT and *mdx4cv* animals will be necessary to ascertain the pathogenic role of the miR-30 family.

**Fig 8:**
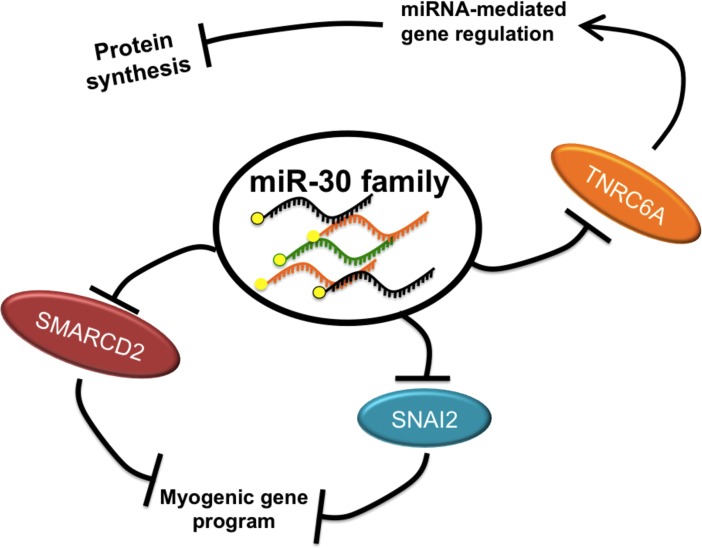
Model for miR-30 family mechanism of action. To promote a myogenic gene program, miR-30 family miRNAs repress the expression of SNAI2 and SMARCD2, both negative regulators of myogenesis. Additionally, miR-30 family miRNAs provide negative feedback on the miRNA pathway by targeting TNRC6A, leading to derepressed miRNA targets and increased protein synthesis.

Transcriptional, post-transcriptional and epigenetic regulation of gene expression are the most highly enriched GO terms in the set of predicted miR-30 family targets. Many of the studies published on various miR-30 family functions indeed report the regulation of transcription factors [26,33,35,39,40], indicating that the generalized function of miR-30 may be to control the switch from one cellular state (i.e. proliferating, differentiating, quiescent, etc.) to another by acting as a global gene expression ‘switch.’ Therefore, by repressing the set of miR-30 targets present in the given cellular milieu while at the same time reducing the extent of other miRNA-mediated repression, miR-30 family can repress a current gene expression pattern and pave the way for a change in cellular state (Fig. 8). In agreement with this argument, the validated miR-30 targets include the epigenetic SWI/SNF component *Smarcd2*, the transcription factor *Snai2*, and the post-transcriptional miRNA pathway component *Tnrc6a*.

TNRC6A is a component of cytoplasmic granules known as P-bodies that are required for miRNA repression [41]. Indeed, P-bodies are enriched for the presence of miRNAs, repressed mRNAs, and argonaute proteins [27]. Importantly, the P-body component GW182 has been shown in *D. melanogaster* to interact with argonaute proteins, and to be required for miRNA repression [42]. In mammals, the GW182 paralogue TNRC6A has been shown to be present in P-bodies and required for efficient miRNA-mediated repression [43–45]. While others have identified *Tnrc6a* as a miR-30 family target [46], we are the first to show that miR-30 expression modulates the activity of other miRNAs and levels of protein synthesis. This observation also raises the intriguing possibility that miR-30 mediated regulation of the miRNA pathway is a mechanism not specific to muscle cells, but rather a mechanism in all cells expressing miR-30 to antagonize the expression of all miRNA-regulated targets.

In conclusion, we present a miRNA-seq dataset identifying a reduction in miR-30 family miRNA expression in dystrophic *mdx4cv* skeletal muscles. We also show that miR-30 family expression is reduced in acute pathological conditions including BaCl_2_-induced injury and disuse atrophy. Through *in vitro* experiments and bioinformatic analysis, we have proposed a novel mechanism whereby miR-30 promotes the differentiation of myoblasts by both restricting the expression of *Smarcd2* and *Snai2* (both negative regulators of the myogenic gene program), as well as by antagonizing the miRNA pathway through repression of *Tnrc6a*. These findings contribute to our understanding of miRNA function during myogenesis.

## Materials and Methods

### Animal care

All animal experiments were performed using protocols approved by the University of Colorado Institutional Animal Care and Use Committees (IACUC). C57Bl/6J animals were obtained from The Jackson Laboratory, and *mdx4cv* (B6Ros.Cg-Dmd^mdx-4Cv^/J) animals were a kind gift from Dr. Jeffrey Chamberlain (University of Washington). Animals were housed under standard conditions in a partial barrier facility and received access to water and chow *ad libidum*. For sample collection, animals were sedated using 1–4% inhaled isoflurane and sacrificed by cervical dislocation.

### Human miR-30 family expression

De-identified, archival DMD biopsy samples were kindly provided by Dr. Eric Hoffman (George Washington University) and have been described previously [47]. Based on the University of Colorado Human Research Institutional Review Board (IRB) guidelines (HRP-310 Worksheet), these samples were considered exempt from IRB approval. Control muscle biopsies were obtained as previously described [47]. Briefly, samples were surgically removed from the vastus lateralis in sedentary, college-aged men in Boulder, Colorado, USA. All control biopsy protocols were approved by the University of Colorado Human Research IRB, Boulder, Colorado, USA (IRB Protocol 0402.11, August 2002). Total RNA was isolated using TRI Reagent (Molecular Research Center), and 7 ng total RNA was reverse transcribed and PCR amplified using Taqman miRNA assays (Invitrogen) using U6 as a reference gene.

### miRNA cloning and sequencing

To prepare the library for Illumina sequencing, small RNAs were isolated from the gastrocnemius muscles of C57Bl/6 and *mdx4cv* mice (n = 2/group) using miRvana miRNA isolation kit (Ambion). 1 μg of small RNA per sample was then adaptor ligated, reverse transcribed and PCR amplified following a modified version of Illumina’s Small RNA Sample Prep v1.5.0. Briefly, small RNAs were first ligated to a 3’ adapter (miRNA Linker 1, IDT) modified with 3’-terminal dideoxy-cytosine and a 5’-adenylation, using T4 RNA Ligase 2, truncated (New England Biolabs) in the absence of ATP (to prevent RNA circularization). The RNAs were then ligated to RNA/DNA hybrid 5’ adapters, using T4 RNA Ligase 1 (New England Biolabs) in the presence of ATP. These 5’ adapters contain a three-nucleotide “barcode” sequence at their 3’ end to mark respective sample libraries. The adapter-ligated RNAs were then reverse transcribed and PCR amplified for a total of 18 cycles using primers complementary to the adapter sequences, with non-complementary overhangs containing the P5/P7 flow cell sequences. The fragments were then gel purified from an 8% polyacrylamide gel and quantified on a 2100 Bioanalyzer (Agilent). Prior to submission, libraries were validated by TOPO-cloning (Invitrogen) and conventional Sanger sequencing to ensure proper adapter orientation and insert identity. Samples were then multiplexed and submitted for single-end (36 cycle) sequencing on an Illumina GAII.

### Sequencing data analysis

Before any downstream analysis, quality assessment of the sequencing run was performed using the SolexaQA software package [48]. Next, the raw fastq file was split into respective biological samples based on the three-nucleotide barcodes present at the 5’ end using the Galaxy server [49]. These barcodes were subsequently trimmed and the files were converted to fasta. The split, trimmed fasta files were then used to generate input files for alignment using the miRanalyzer web tool [50]. Briefly, miRanalyzer uses the short read aligner Bowtie [51] to align short DNA sequences to a given reference genome (miRbase version 16). Following alignment, reads for each sample were normalized to the number of mapped miRNA reads and converted to a percent. C57Bl/6 and *mdx4cv* replicates were then averaged to generate expression values.

### qRT-PCR

First, total RNA was isolated from snap frozen skeletal muscles using TRI Reagent (Molecular Research Center). miRNA expression was measured using Taqman miRNA assays (Invitrogen). Unless indicated, sno202 was used as a reference gene and 7 ng of total RNA per reaction was reverse transcribed. For mRNAs, primer sequences were chosen to amplify unique sequences and span exon-exon junctions (S2 Table). cDNA was produced from 1 μg of total RNA/sample using random hexamers and Superscript III (Life Technologies). Following reverse transcription, cDNA was quantified using SYBR Green. For all experiments, relative expression was determined with 18S as a reference gene, using the E^-ΔΔCt^ method where efficiency (E) is determined using a standard curve (where perfect efficiency = 2). PCR amplification was done on a CFX96 thermocycler (Bio-Rad), with the following cycling parameters: 50°C for 2’, 95°C for 10’ followed by 40 repeated cycles of 95°C for 15” and 60°C for 1’.

### Barium chloride injury

Barium chloride injury was performed as described previously in Guess, et al. [52]. Briefly, mice were anesthetized with 1–4% inhaled isoflurane, and the right hindlimb was shaved. After cleaning the area with 70% ethanol, the right gastrocnemius was injected with 50 μL of a 1.2% barium chloride solution in normal saline using a 27-gauge insulin syringe. Mice recovered on a 37°C heat block and were monitored for adverse effects.

### Hindlimb suspension

Hindlimb suspension was performed as previously described [19]. Briefly, animals (n = 7/group) were individually housed and suspended at 30° head-down using a dowel/swivel apparatus attached to the tail with a biocompatible adhesive, secured to a wire running the length of the cage. To facilitate movement, a 0.25” wire mesh floor was placed in the cage. Mice were monitored for health status and adverse effects of hindlimb suspension. Following sacrifice, wet muscle weights were measured and samples were snap frozen in liquid N_2_.

### Plasmid construction

Full length 3’-UTRs were cloned from C_2_C_12_ genomic DNA with primers (S2 Table) that contained non-complementary Xho1 and Not1 restriction sites using AccuPrime Supermix II (Invitrogen). Following digestion of psiCHECK-2 (Promega) with Xho1/Not1 (New England Biolabs) inserts were gel purified and ligated using T4 DNA ligase (New England Biolabs) downstream of the *Renilla* luciferase coding sequence, before the polyadenylation signal. For miR-206 overexpression, 1 kilobase surrounding the pre-miR-206 locus was amplified from C_2_C_12_ genomic DNA using primers containing HindIII and Xho1 restriction sites (S2 Table), and cloned into pDNA3.1+ (Life Technologies). The resulting plasmids were transformed into DH5α cells, and insert identity was confirmed with Sanger sequencing.

### Cell culture, transfections and luciferase assays

C_2_C_12_ cells were passaged in Dulbecco’s modified Eagle’s medium (DMEM) supplemented with 20% fetal bovine serum (FBS), 2 mM L-glutamine, 1mM sodium pyruvate and 100 U/mL penicillin/streptomycin (Life Technologies). For differentiation, 80–100% confluent cells were switched to DMEM supplemented with 2% horse serum, 2 mM L-glutamine, 1mM sodium pyruvate and 100 U/mL penicillin/streptomycin (Life Technologies). For miRNA knockdown/overexpression, 40–50% confluent cells were transfected with indicated concentrations of antimiRs (miRagen Therapeutics) or pre-miRs (Ambion pre-miR-control [AM17110] or an equimolar mix of pre-miR-30a-5p [PM11062], pre-miR-30b [PM10986] and pre-miR-30c [PM11060]) using Lipofectamine 2000 transfection reagent (Life Technologies) according to the manufacturer’s instructions. For 3’-UTR reporter transfections, 0.5 μg of indicated plasmid reporter construct was transfected along with pre-miRs into 12-well dishes in triplicate. 24 hours following transfection, cells were harvested and luciferase signals were measured using the Dual-Luciferase reporter assay kit (Promega).

### Quantification of differentiation and proliferation

For myogenin quantification, C_2_C_12_ cells were grown on gelatin-coated glass coverslips and transfected with 10nM pre-miRs. 24 hours following transfection, cells were fixed in 4% EM grade paraformaldehyde and stained with 1:50 anti-MYOG antibodies (Santa Cruz SC-576) and 1:200 Texas red-conjugated anti-rabbit secondary antibodies (Jackson 711-075-152) for one hour each at room temperature. For myosin heavy chain (MyHC) quantification, cells were triggered to differentiate 24 hours following transfection of 10nM antimiRs. 48 hours after triggering differentiation, cells were stained with anti-MyHC antibodies (F59 supernatant undiluted, DSHB), and 1:200 FITC-conjugated anti-mouse IgG secondary antibodies (Jackson 715-095-151) for one hour at room temperature. Following antibody staining, all coverslips were then incubated in 300 nM 4',6-diamidino-2-phenylindole (DAPI) for 5 minutes at room temperature (Sigma D-9542) and mounted using Fluoromount-G (Southern Biotech). Images of the stained cells were collected using an EVOS FL fluorescence microscope, and 8–10 non-overlapping images were collected/coverslip. The images were then blindly quantified by counting total DAPI+ nuclei and either MYOG+ nuclei or total MyHC+ area using ImageJ software. Results displayed are representative of 3 independent experiments.

For proliferation assays, cells were electroporated with the indicated concentrations of Ambion pre-miR-control (AM17110) or pre-miR-30a-5p (PM11062) using Amaxa Nucleofector (Lonza AG) and plated on gelatin-coated glass bottom 96-well imaging plates. 24 hours following electroporation, cells were pulsed with 10 μM 5-ethynyl-2’-deoxyuridine (EdU) for 2 hours, then fixed and stained with DAPI and Alexa Fluor 555 according to the Click-iT EdU imaging kit (Life Technologies). Images were collected using an ImageXpress Micro XL (Molecular Devices) automated imaging system. Following imaging, %EdU+ cells was calculated using ImageJ software for 9 images/well for n = 4 wells.

### Protein synthesis

To determine rates of protein synthesis, C_2_C_12_ cells were grown to confluence, then switched to differentiation medium. 48 hours following the induction of differentiation, myotubes were infected with empty adenovirus (control) or adenoviruses encoding the miR-30a hairpin and the miR-30b/d cluster (kind gift of Dr. Eric Olson, UT Southwestern) at a multiplicity of infection (MOI) of 50. Adenovirus production and purification was performed as described previously [53], using the AdEasy Vector System (Qbiogene). Briefly, replication deficient adenoviruses were amplified in HEK293 cells. Viral particles were purified from clarified cell lysates by cesium chloride gradients, and concentrated virus was stored in glycerol at -20°C. 48 hours after infection, cycloheximide (CHX) negative control wells were pre-incubated for one hour with 50 μM CHX to block translation. Following pre-incubation, all wells received 2 μC/mL of ^3^H-tyrosine to label newly synthesized protein (CHX wells received ^3^H-tyrosine in addition to CHX) for 2 hours. Cellular protein was then precipitated in 10% trichloroacetic acid (TCA) for 30 minutes on ice, transferred to 1.5 mL eppendorf tubes, and spun for 5 minutes at full speed. Pellets were resuspended in 0.5 M NaOH + 0.1% Triton X-100 by incubating for 1 hour at 50°C. Scintillation counts were then measured by adding 20 uL of protein sample to 2 mL Ecoscint (National Diagnostics) + 10 uL of 1 N acetic acid. Counts were collected over one minute and normalized to total protein concentration for each sample by bicinchoninic acid (BCA) assay (Pierce).

## Supporting Information

S1 Fig Sequence and organization of miR-30 family miRNAs. **(A)** Alignment of miR-30a-5p, miR-30b, miR-30c, miR-30d and miR-30e shows conserved positions in bold and positions differing from miR-30a-5p in red. Seed sequence is underlined. **(B)** Genomic organization of miR-30 family in mice.
(TIF)

S2 Fig Abundance of miR-30a/b/c in WT skeletal muscle. qRT-PCR measurement of miR-30a/b/c abundance shown for WT soleus, TA, and gastrocnemius muscles relative to sno202. **P ≤0.001 compared to miR-30a-5p, #*P* ≤0.05 and ## P≤0.001 compared to miR-30b. Error bars = SEM.
(TIF)

S3 Fig Muscle characteristics during acute injury and disuse atrophy. H&E stained cryosections from BaCl_2-_injured muscle on indicated days post-injury (DPI). Scale bar = 200μm.
(TIF)

S4 Fig miR-30a/b/c measurement after miR-30a-5p and antimiR-30 transfection. **(A)** qRT-PCR measurement of miR-30a-5p in C_2_C_12_ cells transfected with indicated concentrations of pre-miR-30a-5p or pre-miR-control. **P≤0.001 compared to miR-control transfected cells at equivalent concentrations. Error bars = SEM. **(B)** qRT-PCR measurement of miR-30a/b/c in C_2_C_12_ cells transfected with indicated concentrations of antimiR-30 or antimiR-control. *P≤0.05, **P≤0.001 compared to antimiR-control transfected cells at equivalent concentrations. Error bars = SEM.
(TIF)

S1 Table miRNA-seq expression data. Normalized expression values from miRNA-seq on whole muscle small RNAs isolated from 3-month old *mdx4cv* and C57Bl/6 (WT) gastrocnemius muscles.
(PDF)

S2 Table Primer sequences for qPCR and cloning. (PDF)
