# Supplementary figures and images for: miR-30 Family microRNAs Regulate Myogenic Differentiation and Provide Negative Feedback on the microRNA Pathway
Source: PLoS One. 2015 Feb 17;10(2):e0118229. doi: 10.1371/journal.pone.0118229 (PMC4331529; doi:10.1371/journal.pone.0118229)

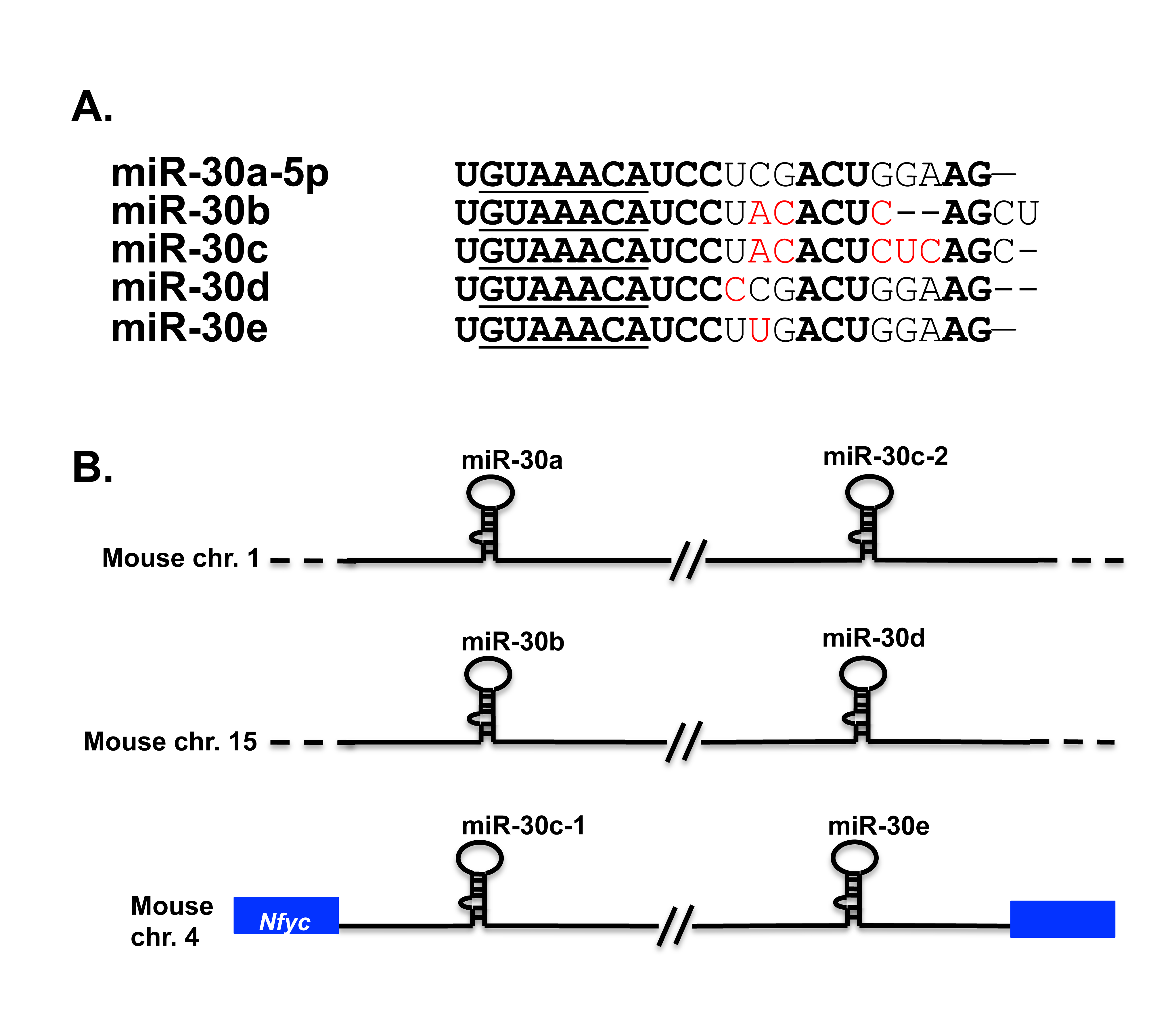

Supplement: S1 Fig — (A) Alignment of miR-30a-5p, miR-30b, miR-30c, miR-30d and miR-30e shows conserved positions in bold and positions differing from miR-30a-5p in red. Seed sequence is underlined. (B) Genomic organization of miR-30 family in mice. (TIF) [file pone.0118229.s001.tif]

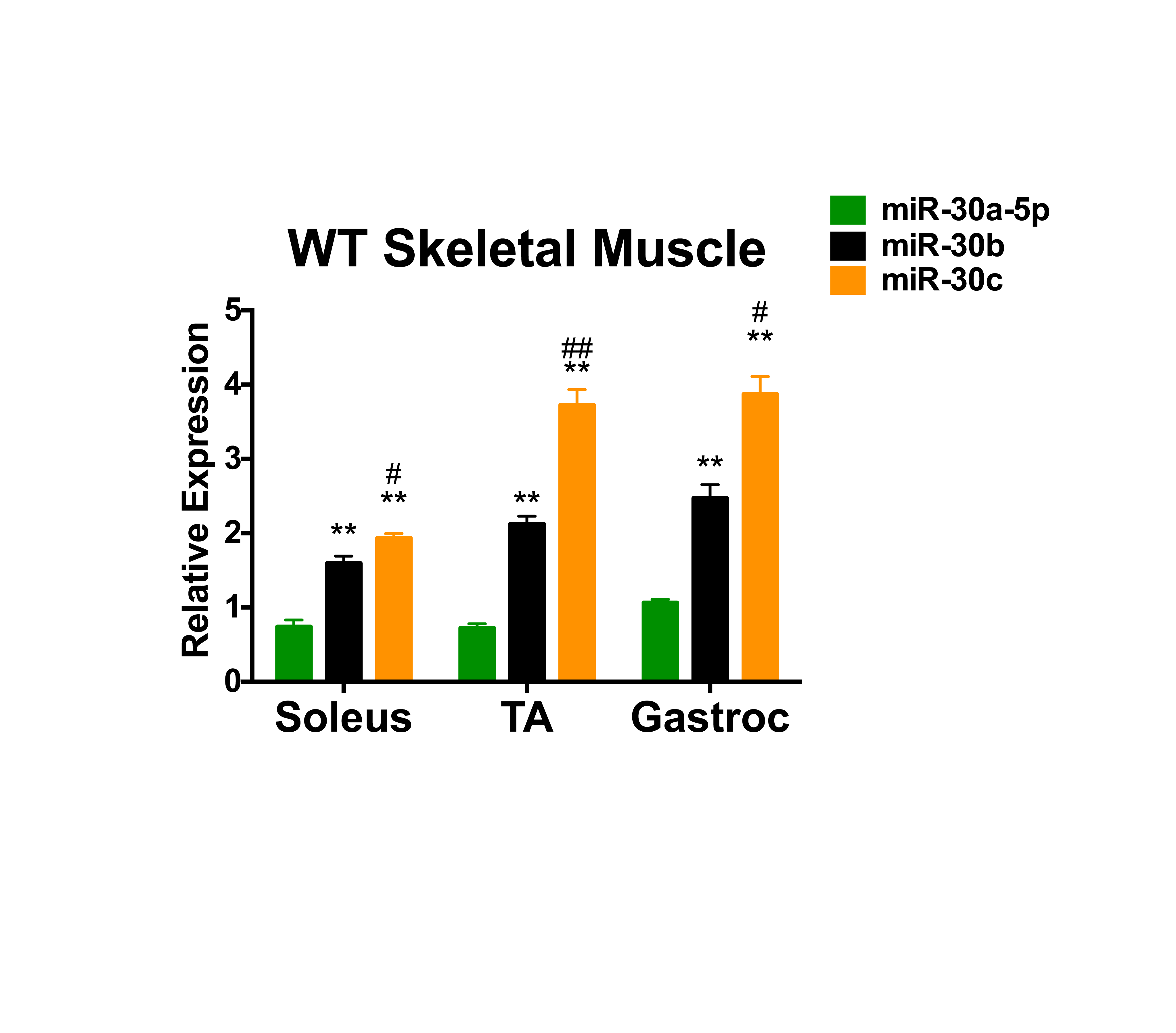

Supplement: S2 Fig — qRT-PCR measurement of miR-30a/b/c abundance shown for WT soleus, TA, and gastrocnemius muscles relative to sno202. **P ≤0.001 compared to miR-30a-5p, #P ≤0.05 and ## P≤0.001 compared to miR-30b. Error bars = SEM. (TIF) [file pone.0118229.s002.tif]

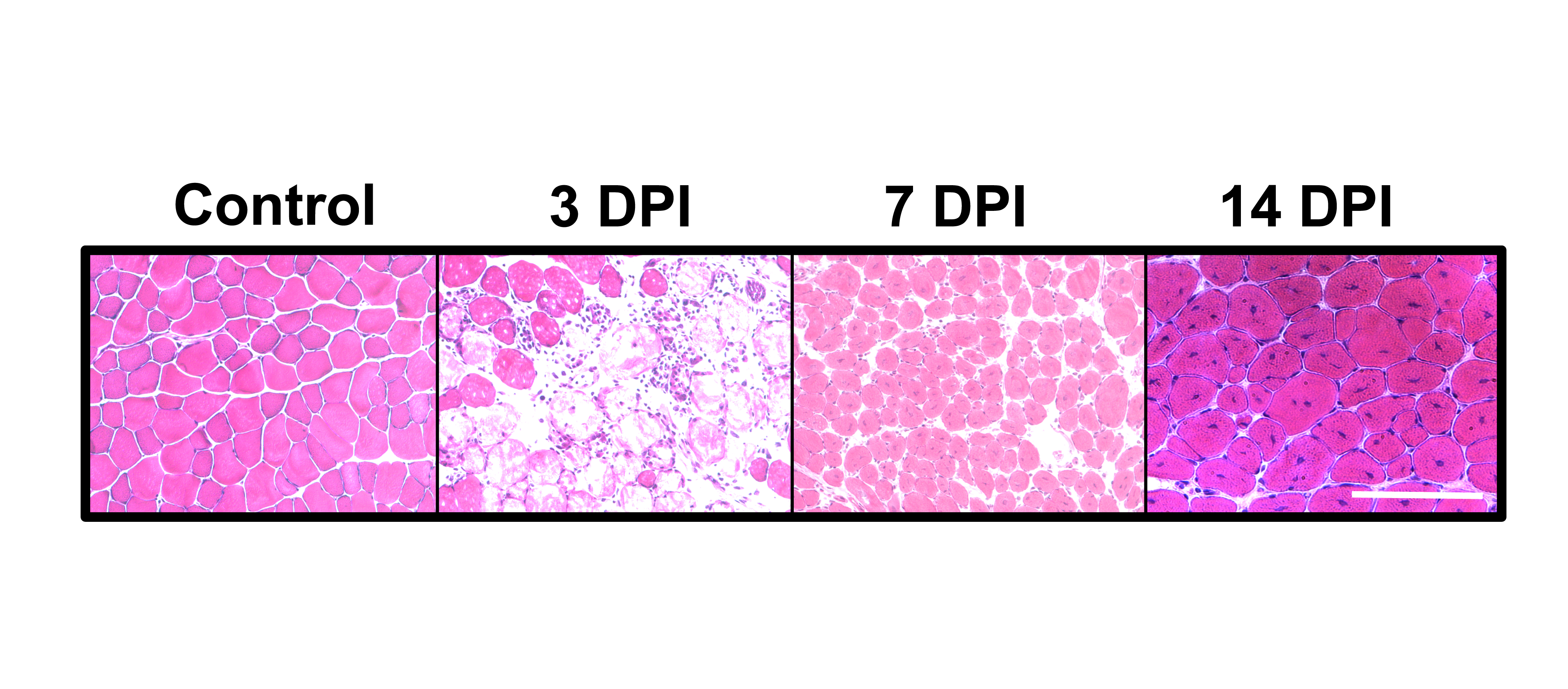

Supplement: S3 Fig — H&E stained cryosections from BaCl2-injured muscle on indicated days post-injury (DPI). Scale bar = 200μm. (TIF) [file pone.0118229.s003.tif]

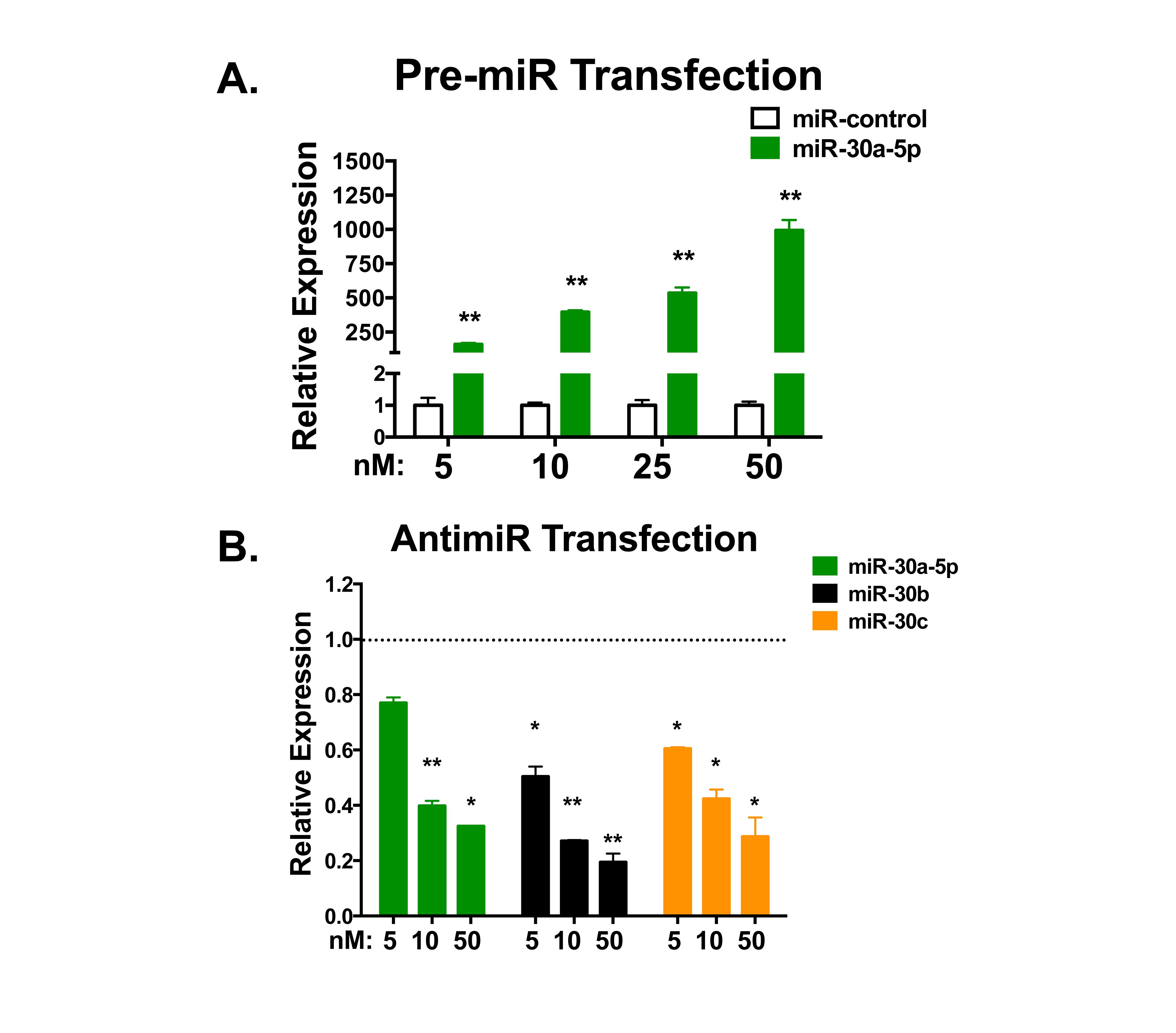

Supplement: S4 Fig — (A) qRT-PCR measurement of miR-30a-5p in C2C12 cells transfected with indicated concentrations of pre-miR-30a-5p or pre-miR-control. **P≤0.001 compared to miR-control transfected cells at equivalent concentrations. Error bars = SEM. (B) qRT-PCR measurement of miR-30a/b/c in C2C12 cells transfected with indicated concentrations of antimiR-30 or antimiR-control. *P≤0.05, **P≤0.001 compared to antimiR-control transfected cells at equivalent concentrations. Error bars = SEM. (TIF) [file pone.0118229.s004.tif]
