# Supplementary material for: miR-30 Family microRNAs Regulate Myogenic Differentiation and Provide Negative Feedback on the microRNA Pathway
Source: PLoS One. 2015 Feb 17;10(2):e0118229. doi: 10.1371/journal.pone.0118229 (PMC4331529; doi:10.1371/journal.pone.0118229)
Supplement: S1 Table — Normalized expression values from miRNA-seq on whole muscle small RNAs isolated from 3-month old mdx4cv and C57Bl/6 (WT) gastrocnemius muscles. (PDF) [file pone.0118229.s005.pdf]

Supplemental Table 1: **miRNA-seq expression data.**

miRNA name	Avg. C57Bl/6	Avg. *mdx4cv*	Fold *mdx4cv*/C57Bl/6
**mmu-miR-1**	37.31043187	28.05404913	0.75190899
**mmu-let-7c**	5.040040343	17.0630017	3.385489112
**mmu-miR-143**	4.107817409	13.55561736	3.299956159
**mmu-miR-21**	1.147044732	9.263272561	8.075772728
**mmu-miR-378**	5.753655759	3.231030682	0.561561348
**mmu-miR-206**	0.456397449	8.393570355	18.39092302
**mmu-miR-26a**	4.363780441	1.115344165	0.255591266
**mmu-let-7f**	2.117834583	3.332810036	1.573687607
**mmu-miR-16**	4.16209549	0.587561826	0.141169713
**mmu-let-7b**	1.555484652	2.709994264	1.742218581
**mmu-miR-133a**	3.304896509	0.950023854	0.287459487
**mmu-miR-24**	2.545681883	1.075113044	0.42232812
**mmu-let-7a**	2.663115793	0.938023867	0.352227969
**mmu-miR-30a**	1.897246362	0.307804182	0.162237329
**mmu-miR-126-3p**	0.945883219	1.166901699	1.233663601
**mmu-miR-196a**	1.724851155	0.375353365	0.217614931
**mmu-miR-29a**	1.824259858	0.213378574	0.116967204
**mmu-miR-27b**	1.710917179	0.257621353	0.150574999
**mmu-let-7i**	1.187277933	0.538210901	0.453315004
**mmu-miR-125b-5p**	0.950867814	0.341467856	0.359111804
**mmu-miR-181a**	0.926728252	0.307226323	0.331517165
**mmu-let-7g**	0.844700099	0.244961435	0.289998113
**mmu-miR-152**	0.677316863	0.333672353	0.492638485
**mmu-miR-22**	0.789658889	0.113121003	0.143252998
**mmu-miR-27a**	0.597730272	0.240490476	0.402339461
**mmu-miR-181b**	0.656599902	0.119801236	0.182456982
**mmu-miR-26b**	0.660251611	0.091507215	0.138594459
**mmu-let-7d**	0.596708143	0.085839487	0.143855063

**mmu-miR-30d**	0.553519225	0.10627255	0.191994325
**mmu-miR-30e**	0.532202786	0.120701133	0.226795379
**mmu-miR-29c**	0.532845961	0.074324744	0.13948636
**mmu-miR-145**	0.189431075	0.41214146	2.175680313
**mmu-miR-195**	0.422513007	0.164419295	0.389146115
**mmu-miR-322**	0.385833845	0.187303614	0.485451486
**mmu-miR-23a**	0.42820001	0.13432223	0.313690394
**mmu-miR-30c**	0.476779302	0.082914891	0.173906231
**mmu-miR-196b**	0.194588823	0.349529864	1.796248417
**mmu-miR-10b**	0.426693863	0.055268618	0.129527568
**mmu-miR-142-3p**	0.039558069	0.440473891	11.13486846
**mmu-miR-101a**	0.099842653	0.324171786	3.24682664
**mmu-miR-100**	0.261653915	0.144618885	0.552710572
**mmu-miR-103**	0.38197825	0.018179099	0.047591974
**mmu-miR-23b**	0.300558766	0.084869442	0.282372207
**mmu-miR-379**	0.157298206	0.225775505	1.435334266
**mmu-miR-126-5p**	0.243751746	0.111588164	0.457794318
**mmu-miR-30a***	0.240727442	0.053043957	0.220348609
**mmu-miR-199b**	0.184329813	0.098335002	0.533473127
**mmu-miR-139-5p**	0.242471136	0.012092881	0.049873487
**mmu-miR-15a**	0.193215402	0.058494237	0.302741066
**mmu-miR-101b**	0.105001673	0.118274692	1.126407686
**mmu-miR-140***	0.093691663	0.048498376	0.517638119
**mmu-miR-10a**	0.079874018	0.059953978	0.750606758
**mmu-let-7e**	0.086737102	0.050945341	0.58735351
**mmu-miR-93**	0.096807112	0.03684468	0.380598897
**mmu-miR-30b**	0.087622145	0.027232083	0.310789961
**mmu-miR-128**	0.044191147	0.070392835	1.59291712
**mmu-miR-99a**	0.034548262	0.062676462	1.814171196

**mmu-miR-191**	0.050165627	0.046265887	0.922262705
**mmu-miR-181d**	0.052959381	0.041077398	0.775639689
**mmu-miR-214**	0.077634873	0.012056591	0.155298649
**mmu-miR-125a-5p**	0.064900126	0.022786227	0.351096808
**mmu-miR-130a**	0.076130313	0.010581806	0.138995964
**mmu-miR-98**	0.059830268	0.022445687	0.375156054
**mmu-miR-652**	0.051808554	0.029550565	0.570380041
**mmu-miR-148b**	0.076514038	0.003449502	0.045083254
**mmu-miR-30e***	0.051624715	0.026773018	0.518608528
**mmu-miR-425**	0.046060717	0.030941505	0.671754736
**mmu-miR-15b**	0.070060739	0.005396263	0.077022633
**mmu-miR-361**	0.069092394	0.004861126	0.070356884
**mmu-miR-151-3p**	0.045918136	0.02476229	0.539270371
**mmu-miR-676**	0.04044515	0.029121062	0.720013684
**mmu-miR-29b**	0.054913056	0.014107905	0.256913498
**mmu-miR-19b**	0.034321436	0.034145063	0.99486113
**mmu-miR-486***	0.050530267	0.016195524	0.320511346
**mmu-miR-872**	0.047548916	0.01800331	0.378627149
**mmu-miR-194**	0.054658164	0.010275667	0.187998764
**mmu-miR-185**	0.048556943	0.011819807	0.243421563
**mmu-miR-99b**	0.032303324	0.020375814	0.630765231
**mmu-miR-25**	0.030538975	0.019127614	0.626334504
**mmu-miR-221**	0.026308472	0.02310687	0.878305307
**mmu-miR-324-5p**	0.044390557	0.003086114	0.069521863
**mmu-miR-127**	0.023082672	0.021924249	0.94981417
**mmu-miR-22***	0.042573182	0.001597349	0.037520085
**mmu-miR-7a-1***	0.040777974	0.003307586	0.081112078
**mmu-miR-222**	0.032442245	0.011633807	0.358600565
**mmu-miR-155**	0.029649564	0.013768711	0.464381576

**mmu-miR-223**	0.005517081	0.036982017	6.703184845
**mmu-miR-92a**	0.027074046	0.0116975	0.43205586
**mmu-miR-28**	0.009489233	0.02897215	3.053160397
**mmu-miR-497**	0.019500546	0.018941886	0.97135159
**mmu-miR-542-3p**	0.000537864	0.037514245	69.74671197
**mmu-miR-378***	0.026119293	0.011722765	0.448816332
**mmu-miR-18a**	0.02694577	0.01083956	0.402273172
**mmu-miR-199a-5p**	0.023778446	0.011780558	0.495430101
**mmu-miR-1843-5p**	0.027334687	0.006182172	0.226165818
**mmu-miR-34a**	0.00651955	0.025805675	3.95819914
**mmu-miR-148a**	0.00848222	0.022942663	2.704794593
**mmu-miR-107**	0.030949809	0.000373871	0.012079905
**mmu-miR-186**	0.028495937	0.002371432	0.083219997
**mmu-miR-342-3p**	0.018761261	0.009791324	0.521890512
**mmu-miR-149**	0.024477741	0.003776335	0.154276301
**mmu-miR-423-3p**	0.014841423	0.012771539	0.860533318
**mmu-miR-190**	0.021647191	0.005109744	0.236046525
**mmu-miR-122**	0.011456415	0.015236258	1.329932385
**mmu-miR-151-5p**	0.012870888	0.01190312	0.924809602
**mmu-miR-193***	0.019667873	0.002897699	0.147331602
**mmu-miR-181c**	0.014722598	0.007642712	0.519114382
**mmu-miR-29a***	0.017430555	0.004828602	0.277019426
**mmu-miR-338-3p**	0.019546792	0.001514566	0.077484115
**mmu-miR-28***	0.015456453	0.005459695	0.353230806
**mmu-miR-17**	0.011737045	0.008396634	0.715395926
**mmu-miR-146b**	0.004536358	0.015248077	3.361303404
**mmu-miR-451**	0.010457726	0.008970477	0.857784613
**mmu-miR-7a**	0.004414106	0.014792488	3.351185704
**mmu-miR-150**	0.01394619	0.004196973	0.300940505

**mmu-miR-744**	0.01332571	0.004802531	0.360395901
**mmu-miR-1839-5p**	0.011323988	0.006794718	0.600028695
**mmu-miR-532-5p**	0.006624016	0.010791454	1.629140516
**mmu-miR-301a**	0.010137279	0.007161061	0.706408536
**mmu-miR-20a**	0.01134353	0.005587363	0.492559429
**mmu-miR-143***	0.015228096	0.000555833	0.036500524
**mmu-miR-30c-2***	0.011492413	0.003766122	0.327705087
**mmu-miR-140**	0.006946805	0.007411025	1.066824931
**mmu-miR-34c**	0.000342831	0.013915997	40.59147268
**mmu-miR-541**	0.007177963	0.006802782	0.947731519
**mmu-miR-19a**	0.004925307	0.008739865	1.774481133
**mmu-miR-133b**	0.002710905	0.010725329	3.956364562
**mmu-miR-423-5p**	0.008758092	0.004399099	0.502289624
**mmu-miR-365**	0.00790921	0.004553911	0.575773121
**mmu-miR-136**	0.004699652	0.007463975	1.588197141
**mmu-miR-484**	0.009633816	0.002412555	0.250425654
**mmu-miR-31**	0.000522314	0.011345675	21.72195578
**mmu-miR-382**	0.00535945	0.006248828	1.165945771
**mmu-miR-1198-5p**	0.010050385	0.001540369	0.153264656
**mmu-miR-411***	0.000580183	0.010680186	18.4083056
**mmu-miR-146a**	0.00021101	0.010812966	51.24397558
**mmu-miR-331-3p**	0.008122562	0.002709016	0.333517483
**mmu-miR-434-3p**	0.009380881	0.00138179	0.147298517
**mmu-miR-183**	0.007618959	0.002496414	0.327658132
**mmu-miR-376b***	0.005450282	0.004515481	0.828485721
**mmu-miR-1959**	0.004214368	0.005598654	1.328468218
**mmu-miR-335-5p**	0.002110201	0.007509402	3.558618689
**mmu-miR-138**	0.006373402	0.003031819	0.475698653
**mmu-miR-434-5p**	0.007745863	0.00156644	0.202229215

**mmu-miR-132**	0.005443076	0.003807245	0.699465641
**mmu-miR-337-5p**	0.003612387	0.005411051	1.497915658
**mmu-miR-218**	0.004519797	0.004474086	0.989886624
**mmu-miR-193b**	0.006530981	0.002135444	0.326971357
**mmu-miR-324-3p**	0.006847948	0.001097691	0.16029485
**mmu-miR-1981**	0.005485502	0.002145121	0.391052837
**mmu-miR-125b-2-3p**	0.004414106	0.002792338	0.632594227
**mmu-miR-144**	0.005134008	0.001869622	0.364164177
**mmu-miR-574-5p**	0.004484311	0.002490502	0.555381071
**mmu-miR-542-5p**	0.004140044	0.00274557	0.663174298
**mmu-miR-106b**	0.002136012	0.004741518	2.219799157
**mmu-miR-299***	0.003116522	0.003619638	1.161434869
**mmu-miR-501-3p**	0.001054888	0.005607253	5.315497361
**mmu-miR-204**	0.005700896	0.000803378	0.140921423
**mmu-miR-142-5p**	0.000253169	0.006190238	24.45102668
**mmu-miR-674**	0.002622095	0.003357311	1.280392458
**mmu-miR-101a***	0.001239661	0.004471933	3.607384151
**mmu-miR-186***	0.004250545	0.001213265	0.285437588
**mmu-miR-362-5p**	0.000970569	0.004469246	4.604767709
**mmu-miR-125b-1-3p**	0.00202551	0.003356235	1.656982527
**mmu-miR-409-3p**	0.000970569	0.00434937	4.481257157
**mmu-miR-182**	0.004355012	0.000862778	0.198111576
**mmu-miR-9**	0.003333514	0.001753241	0.525943905
**mmu-miR-455**	0.003740461	0.001249282	0.333991327
**mmu-miR-411**	0.002996399	0.00194273	0.648354916
**mmu-miR-340-3p**	0.001851211	0.002930758	1.583157769
**mmu-miR-3068***	0.001929654	0.002823251	1.463087018
**mmu-miR-328**	0.004013938	0.000702318	0.174969762
**mmu-miR-33***	0.004503874	0.000202121	0.044877203

**mmu-miR-29b-1***	0.003339495	0.001324003	0.396468106
**mmu-miR-543**	0.000664608	0.003991895	6.006389466
**mmu-miR-203**	0.002610185	0.002018257	0.773223982
**mmu-miR-376c**	0.002568398	0.001768561	0.688585282
**mmu-miR-29c***	0.00377632	0.000546964	0.144840462
**mmu-miR-32**	0.00151383	0.002763848	1.82573236
**mmu-miR-154**	0.000216299	0.003818802	17.655168
**mmu-miR-340-5p**	0.001519173	0.002330309	1.533932956
**mmu-miR-664**	0.00311102	0.000711187	0.228602675
**mmu-miR-483**	0.00056939	0.003131806	5.500279286
**mmu-miR-322***	0.002801685	0.000890463	0.317831213
**mmu-miR-450a**	0.000253062	0.003421549	13.52057749
**mmu-miR-379***	0.000506391	0.003162446	6.245068321
**mmu-miR-300**	0.002774543	0.000879174	0.316871563
**mmu-let-7d***	0.002615954	0.000940187	0.359405219
**mmu-miR-500**	0.001741188	0.00168954	0.970337945
**mmu-miR-351**	0.001730342	0.001645998	0.951256139
**mmu-miR-674***	0.001956316	0.001414849	0.723220984
**mmu-miR-193**	0.00242637	0.000942337	0.388373125
**mmu-miR-192**	0.001313027	0.001850003	1.408960173
**mmu-miR-421**	0.0022572	0.000859015	0.380566757
**mmu-miR-224**	0.000680478	0.002425455	3.564341873
**mmu-miR-127***	0.001782549	0.001307876	0.733711166
**mmu-miR-374**	0.001566462	0.001508921	0.96326706
**mmu-miR-299**	0.001829785	0.001208159	0.660273686
**mmu-miR-139-3p**	0.002062593	0.000963571	0.467164993
**mmu-miR-1306**	0.00206735	0.000958732	0.463749371
**mmu-miR-144***	0.002805165	0.000195133	0.069562097
**mmu-miR-574-3p**	0.000975806	0.001974983	2.023950941

**mmu-miR-335-3p**	0.000885718	0.002056423	2.321757488
**mmu-miR-708**	0.001898234	0.001034528	0.544994919
**mmu-miR-503**	0.001461303	0.001429901	0.978510573
**mmu-miR-341**	0.001423742	0.001445223	1.015087462
**mmu-miR-134**	0.000464019	0.002351811	5.068354716
**mmu-miR-669c**	0.00056971	0.002223334	3.902573445
**mmu-miR-135a**	0.001545782	0.001143114	0.739505757
**mmu-miR-532-3p**	0.000944173	0.001743296	1.846373293
**mmu-miR-433**	0.002200236	0.000445903	0.202661573
**mmu-miR-320**	0.001524675	0.001083177	0.710431005
**mmu-miR-210**	0.001551072	0.001043397	0.672694597
**mmu-miR-671-5p**	0.000601023	0.001920152	3.194805484
**mmu-miR-208b**	0.001266057	0.001207353	0.953632441
**mmu-miR-33**	0.002274134	0.000182501	0.08025057
**mmu-let-7i***	0.001604023	0.000738872	0.460636584
**mmu-miR-339-3p**	0.001223791	0.001111667	0.90837956
**mmu-miR-1944**	0.000833245	0.001435007	1.722190674
**mmu-miR-345-5p**	0.002062699	0.000202121	0.097988732
**mmu-miR-431**	0.000664502	0.001599231	2.406662147
**mmu-miR-214***	0.000537917	0.001635785	3.040960514
**mmu-miR-212-3p**	0.001075728	0.001097691	1.020416642
**mmu-miR-877**	0.000812299	0.001357332	1.670976849
**mmu-miR-200c**	0.001228496	0.000812786	0.661610724
**mmu-miR-148b***	0.000817323	0.001212728	1.483780963
**mmu-miR-133a***	0.001371482	0.000612008	0.446238664
**mmu-miR-10b***	0.000838376	0.001036679	1.236533257
**mmu-miR-190b**	0.000722743	0.001140695	1.578284874
**mmu-miR-672**	0.001371748	0.00048622	0.354453084
**mmu-miR-10a***	0.001423476	0.000373871	0.262646363

**mmu-miR-188-5p**	0.000253275	0.001541175	6.084978685
**mmu-miR-499**	0.00072269	0.001058987	1.465340404
**mmu-miR-511-3p**	0.000490521	0.001274547	2.598351957
**mmu-miR-3096-5p**	0.001265312	0.000480038	0.379383151
**mmu-miR-350***	0.000960096	0.00078322	0.81577257
**mmu-miR-338-5p**	0.001329482	0.000404243	0.304060109
**mmu-miR-17***	0.001524888	0.000202121	0.132548243
**mmu-miR-369-3p**	0.001039125	0.000658508	0.633713832
**mmu-miR-184**	0.000885772	0.000757955	0.855700048
**mmu-miR-132***	0.000511574	0.001061137	2.074257143
**mmu-miR-361***	0.000770139	0.00078322	1.016984529
**mmu-miR-720**	0.001018338	0.000530568	0.521014096
**mmu-miR-339-5p**	0.000801613	0.00073269	0.914019584
**mmu-miR-483***	6.33E-05	0.001465379	23.16233976
**mmu-miR-455***	0.000474758	0.001020014	2.14849168
**mmu-miR-495**	0.000469521	0.001014907	2.161577943
**mmu-miR-676***	0.000775323	0.000697211	0.899252711
**mmu-miR-3061-5p**	0.001234371	0.000202121	0.16374434
**mmu-miR-27b***	0.000928676	0.000498853	0.537165714
**mmu-miR-191***	0.001371642	5.05E-05	0.0368393
**mmu-miR-345-3p**	0.001102337	0.000303182	0.275035572
**mmu-miR-29b-2***	0.000801825	0.000581099	0.724719605
**mmu-miR-24-2***	0.000126638	0.001248746	9.860779681
**mmu-miR-145***	0.000833192	0.000534869	0.641951755
**mmu-miR-496**	0.001139685	0.000227386	0.199516815
**mmu-miR-376a**	0.000200377	0.001136932	5.673978478
**mmu-miR-200b**	0.000796216	0.000516592	0.648808801
**mmu-miR-106b***	0.000849434	0.000460417	0.542028144
**mmu-miR-493***	0.001171105	0.000126326	0.107868848

**mmu-miR-1199**	0.001186762	7.96E-05	0.067038249
**mmu-miR-1943**	0.001007439	0.000253189	0.251319956
**mmu-miR-99b***	0.000353357	0.000884281	2.502512507
**mmu-miR-487b**	0.001034154	0.000202121	0.195445975
**mmu-miR-30b***	0.000511681	0.000720595	1.408289963
**mmu-miR-330**	0.000802038	0.000429508	0.535520126
**mmu-miR-124**	0.000965439	0.000252652	0.261696058
**mmu-miR-1839-3p**	0.000796163	0.000404243	0.507738391
**mmu-miR-539-5p**	0.000970782	0.000217173	0.223709511
**mmu-miR-301b**	7.38E-05	0.001111667	15.05398879
**mmu-miR-380-3p**	6.33E-05	0.001086402	17.17207947
**mmu-miR-467c**	0.000253222	0.000884281	3.492115071
**mmu-miR-342-5p**	0.000358753	0.000778113	2.168936254
**mmu-miR-701**	0.000669845	0.00046418	0.692967007
**mmu-miR-377**	0.000675401	0.000454773	0.673337805
**mmu-miR-501-5p**	0.000232063	0.000888044	3.826742017
**mmu-miR-350**	0.000448255	0.000666302	1.486432615
**mmu-miR-381**	0.000685927	0.000420638	0.613240088
**mmu-miR-199b***	0.000706714	0.00038274	0.541577295
**mmu-miR-196a-2***	0.000580023	0.000462299	0.797034871
**mmu-miR-337-3p**	0.000632869	0.000404243	0.638746147
**mmu-miR-664***	0.00056955	0.000457192	0.802725271
**mmu-miR-1843-3p**	0.000664555	0.000357475	0.537916759
**mmu-miR-125a-3p**	0.00053284	0.000480038	0.900904179
**mmu-miR-30d***	0.000690898	0.000303182	0.438823089
**mmu-miR-3068**	0.000458995	0.000496434	1.081566616
**mmu-miR-467a**	9.49E-05	0.000859015	9.05194887
**mmu-miR-3057-5p**	0.000796269	0.000151591	0.190376443
**mmu-miR-547**	0.00039028	0.000474931	1.216899865

**mmu-miR-675-3p**	0.000126638	0.00073269	5.785717438
**mmu-miR-30c-1***	0.000400966	0.000450204	1.122798766
**mmu-miR-380-5p**	0.000690951	0.000155354	0.224840732
**mmu-miR-505-5p**	0.000342831	0.000497509	1.45118121
**mmu-miR-374c***	0.000479835	0.000353712	0.737153764
**mmu-miR-582-5p**	9.49E-05	0.00073269	7.720779919
**mmu-miR-99a***	0.000654028	0.000167987	0.256848978
**mmu-miR-369-5p**	0.000121295	0.000675171	5.566377311
**mmu-miR-27a***	0.000379913	0.000410425	1.080312998
**mmu-miR-300***	0.000379913	0.000404243	1.064039989
**mmu-miR-669o-5p**	0.000295488	0.000480038	1.624561054
**mmu-miR-1983**	6.33E-05	0.000707424	11.18181919

Raw read counts from miRNA-seq experiment were normalized to the total number of miRNA reads/sample, then averaged for n = 2 animals for WT and *mdx4cv* groups. Values displayed are percentage of total reads for that sample. Top 300 most abundantly cloned miRNAs are displayed.
