# Supplementary material for: miR-30 Family microRNAs Regulate Myogenic Differentiation and Provide Negative Feedback on the microRNA Pathway
Source: PLoS One. 2015 Feb 17;10(2):e0118229. doi: 10.1371/journal.pone.0118229 (PMC4331529; doi:10.1371/journal.pone.0118229)
Supplement: S2 Table — (PDF) [file pone.0118229.s006.pdf]

Supplemental Table 2: Primer sequences for qPCR and cloning

Gene	Primer Use	Direction	Sequence
mmu-miR-206	cloning	Forward	TAGTAAGCTTCCTGCACCCTGGTAATTATAGTG
mmu-miR-206	cloning	Reverse	TAGTCTCGAGCACAGGGCACTGGGTTGAGAAC
Ccnd1	3'-UTR	Forward	ATTCTCGAGGGGCCACCGGGCAGG
Ccnd1	3'-UTR	Reverse	ATTTGCGGCCGCTGAGATTTTACCAATTT
Nfyb	qPCR	Forward	GATCCAGCCCCATGATGATA
Nfyb	qPCR	Reverse	TGAAACTTTCTTTTGAACCATTTG
Ppargc1a	qPCR	Forward	GATCCTCGAGTCGGAAGGACGGACGGAACCGGGC
Ppargc1a	qPCR	Reverse	GATCGCGGCCGCCCTTCCTGCTGTGTATCTGGCAACC
Runx1	qPCR	Forward	CTCCGTGCTACCCACTCACT
Runx1	qPCR	Reverse	ATGACGGTGACCAGAGTGC
Runx1	3'-UTR	Forward	CCTACTCGAGCTGAGCGCCATCGCCATCG
Runx1	3'-UTR	Reverse	CCTAGCGGCCGCTAGGTGCTTGTCAAATTGTTTATTTG
Smarcd2	qPCR	Forward	TCCTGGGAGCTTCGAGTAGA
Smarcd2	qPCR	Reverse	CACAAGGCTCTTAAAGAATGATGA
Smarcd2	3'-UTR	Forward	GATCCTCGAGCTGCTCAGGGATTGCCTCCTTCCTTCCT
Smarcd2	3'-UTR	Reverse	GATCGCGGCCGCACCGACTGCAAAAGGTAACAGCAGTCCTC
Snai2	qPCR	Forward	CATTGCCTTGTGTCTGCAAG
Snai2	qPCR	Reverse	CAGTGAGGGCAAGAGAAAGG
Snai2	3'-UTR	Forward	GATCCTCGAGGTGGCGCAACCAGTGTTTACTC
Snai2	3'-UTR	Reverse	GATCGCGGCCGCAGGCGTGGCTATTAACCGTACC
Tnrc6a	qPCR	Forward	ACAAGCCAATTGGTTATTCTCC
Tnrc6a	qPCR	Reverse	GAAATTCTGATGCCAGATATACCC
Tnrc6a	3'-UTR	Forward	GATCCTCGAGTGGCATAGGTATAGACGTAAACTGT
Tnrc6a	3'-UTR	Reverse	GATCGCGGCCGCGATTTGAAGTCATACAGGTTCT
Galnt7	qPCR	Forward	GACAAAAACTGAGCCGTATCG
Galnt7	qPCR	Reverse	TTCTCTATGGCAAACAATCCAC
Galnt7	3'-UTR	Forward	GATCCTCGAGCTGCTCAGGGATTGCCTCCTTCCTTCCT
Galnt7	3'-UTR	Reverse	GATCGCGGCCGCACCGACTGCAAAAGGTAACAGCAGTCCTC
